# Allergen Tests of Fruit Sensitization Involving Children with Allergic Diseases

**DOI:** 10.3390/children9040470

**Published:** 2022-03-28

**Authors:** Ling-Sai Chang, Hsin-Yu Chang, Yao-Hsu Yang, Zon-Min Lee, Mindy Ming-Huey Guo, Ying-Hsien Huang, Ho-Chang Kuo

**Affiliations:** 1 Department of Pediatrics and Kawasaki Disease Center, Kaohsiung Chang Gung Memorial Hospital, Kaohsiung 833, Taiwan; joycejohnsyoko@gmail.com (L.-S.C.); b9402050@cgmh.org.tw (H.-Y.C.); mindymhguo@yahoo.com.tw (M.M.-H.G.); yhhuang123@yahoo.com.tw (Y.-H.H.); 2 College of Medicine, Chang Gung University, Taoyuan 333, Taiwan; 3 Health Information and Epidemiology Laboratory, Chiayi Chang Gung Memorial Hospital, Chiayi 613, Taiwan; r95841012@ntu.edu.tw; 4 Department of Traditional Chinese Medicine, Chiayi Chang Gung Memorial Hospital, Chiayi 613, Taiwan; 5 School of Traditional Chinese Medicine, College of Medicine, Chang Gung University, Taoyuan 333, Taiwan; 6 Department of Pharmacy, Kaohsiung Chang Gung Memorial Hospital, Kaohsiung 833, Taiwan; zonmin@cgmh.org.tw

**Keywords:** dermatitis, fruits, immunoglobulin E, kiwi, pineapple

## Abstract

Fruit is a kind of plant food which is rich in nutrients and immune-regulating ingredients. A meta-analysis has demonstrated that fruits have a protective effects against asthma. On the other hand, clinical syndromes of allergic reactions to fruits manifest as an oral allergy syndrome. We aimed to investigate the patterns and associated factors of fruit allergen-specific IgE (sIgE) sensitization among patients with suspected clinical symptoms. Data were extracted from the Chang Gung Research Database. Fruit sensitization in Taiwan was evaluated using the presence of IgE antibodies against specific fruits. The overall prevalence of positive sIgE responses to fruit allergens in Taiwan, in order of decreasing importance, was pineapple, kiwi, banana, and papaya. Children aged 0–18 had a higher positive rate of allergic responses to pineapple, kiwi, banana, and papaya than adults over the age of 18. Positive specific IgE for kiwi, banana, or papaya was more frequent in younger than in older children and children with a higher total IgE of both logarithmic (log) and arithmetic values. The analysis of log IgE for pineapple positive vs. negative children determined an optimal cutoff value, log IgE 2.2, with both sensitivity (0.9) and specificity (0.5). Dermatitis was significantly more prevalent in children with positive IgE for pineapple, kiwi, banana, and papaya than negative specific IgE. The highest positive rate of sIgE against fruits was pineapple among children. Even in older children, the positive rate of pineapple allergens was high. IgE discriminates with and without sIgE for pineapple, with an optimal cutoff of 158.5 U/mL.

## 1. Introduction

Allergies constitute a global public health burden, and food allergies have increased worldwide [1,2,3]. The top-ranking causative allergies of food in children include cow’s milk and egg whites [4]. Common allergies are further complicated by population, geography, age, ingested quantities, and dietary exposure patterns [3]. The cause of food allergies mainly prevalent in infants and preschool children is primarily immunoglobulin E (IgE)-mediated adverse reactions following the ingestion of causative food proteins. A questionnaire survey in South Korea, a neighboring Asian country, found that in children younger than six years old, fruit is the most common food type that causes allergic symptoms [5]. The most common fruits triggering anaphylaxis in Korean children were kiwi and peach [6]. Allergic reactions to fruits were usually mild, such as skin reactions [7]. Food allergies in infants have been associated with fruit through a questionnaire from prospective German birth cohorts [8]. Similarly, in Kim’s Korean birth cohort, fruits were identified as one of the common food allergens in infants [6].

It has been argued, by Hua et al., that the diversity of allergenic foods in infancy, including fruits, may protect infants from IgE sensitization at the age of one year [9]. Fresh fruits are key components of healthy dietary patterns inversely associated with impaired renal function in Taiwanese adults with metabolic syndrome [10]. Furthermore, they are popular foods due to their high vitamin, polyphenols, flavonoids, and flavonol contents and thus have perceived health benefits of restraining histamine release, interleukin 4 production, and specific IgE (sIgE) formation [11,12]. Fruit extracts have also been found to exert immunomodulatory, protective effects against allergies, cancers, and inflammation [13]. Statistics have indicated that fruit intake reduced the risk and severity of asthma in children [14]. However, if the fruit is not heated during the process of eating, sensitization to these heat-labile and digestible proteins becomes a concern [15].

Challenge tests found that the prevalence of allergy to fruits greater than 1% included apples, pineapples, and kiwi, and the skin prick tests found only apples to be greater than 1% [16]. The prevalence of allergy to fruit was estimated to be between 0.1 and 4.3% [16]. However, advising parents on the risks of food allergies associated with fruits would alter children’s dietary habits. Research on the patterns of allergen sensitization is vital for providing an updated epidemiology basis for preventing allergic diseases and helping clinicians offer individualized management for unique patient groups.

Data about fruit sensitization in children, especially tropical fruits, is scarce. In vitro testing of serum allergens in patients is an important method for identifying allergens. Although the number of hospital visits for patients with suspected allergy symptoms has decreased and dietary habits have also changed during the COVID-19 pandemic, the positive rate of food sensitization has not changed [17,18]. While hospital-based studies may report some practical information, large-scale all-age studies of IgE-mediated fruit sensitization have not yet been performed in Taiwanese children. Therefore, in this study, we sought to explore the results of sIgE tests against fruits commonly indicated for patients with allergic diseases in Taiwan.

## 2. Materials and Methods

We adopted a multi-center hospital-based (four branches including two tertiary medical centers) approach. Specific IgE was retrospectively collected from an administrative database. All fruits in the Chang Gung Research Database (CGRD) were analyzed. In CGRD, testing for allergen fruit was entirely performed at the discretion of the individual clinician. Data consisted of all age groups of patients with allergic diseases who had asthma, dermatitis, or rhinitis and higher IgE, eosinophil counts, or positive phadiatop allowed by insurance claims during the period 2015–2019 in Taiwan. The guidelines for sIgE at the Chang Gung Memorial Hospital (CGMH) followed the rules established by Taiwan Health Insurance, which do not support asymptomatic sIgE testing. Specific IgE data have only been integrated with CGMH since 2015. Information on the patients’ age, sex, total IgE, and diagnosis was also obtained. We further analyzed the positive rate of various fruit allergens and the associated factors of specific allergens. The numbers of patients investigated and the positive percentage were listed in the Supplementary Table S1. Whole fresh fruits of pineapple, kiwi, banana, papaya, coconut, grapefruit, plum, peach, lemon, strawberry, pear, watermelon, apple, citrus, cherry, orange, mango, melon, or grape were used for sensitization tests. The research protocol was approved by the Ethics Committee of the Chang Gung Memorial Hospital (201900878B0C101).

Specific IgE levels of patients’ sera samples were measured against allergens according to the standardized procedure guidelines (Thermo Fisher Scientific Inc, Phadia AB, Uppsala, Sweden). In CGRD, the test (immunoCAP) identified allergen-sIgE against fruits in serum [19]. The sIgE levels were defined as positive results for values ≥0.35 U/mL, and a detected content under 0.35 U/mL was considered negative reactivity [20].

The demographic information on patients and allergic comorbidities (asthma, dermatitis, and rhinitis established on the basis of the International Classification of Disease codes) were compared between the negative and positive sIgE groups for fruits. All allergic illnesses were followed at the outpatient clinics, inpatient ward and/or emergency rooms. We defined the seasons as the time of sIgE determination: spring as March–May; summer as June–August; autumn as September–November; and winter as December–February [21].

To obtain the best cutoff value of pediatric logarithmic (log) IgE, which was the coordinate point corresponding to the point closest to the upper left corner of the receiver operating characteristic curve, we selected the best critical point according to the largest Youden’s index [22]. Statistical analyses were performed by applying SAS statistical software (Version 9.4; SAS Institute, Cary, NC, USA) to the anonymized data file. Differences in the total IgE and age as continuous variables were tested using t-tests, and differences in the sex and sIgE as categorical variables were tested using χ^2^ tests. *p* values <0.05 were defined as statistically significant. Continuous variables were presented as the mean ± standard deviation.

## 3. Results

**Figure 1:**
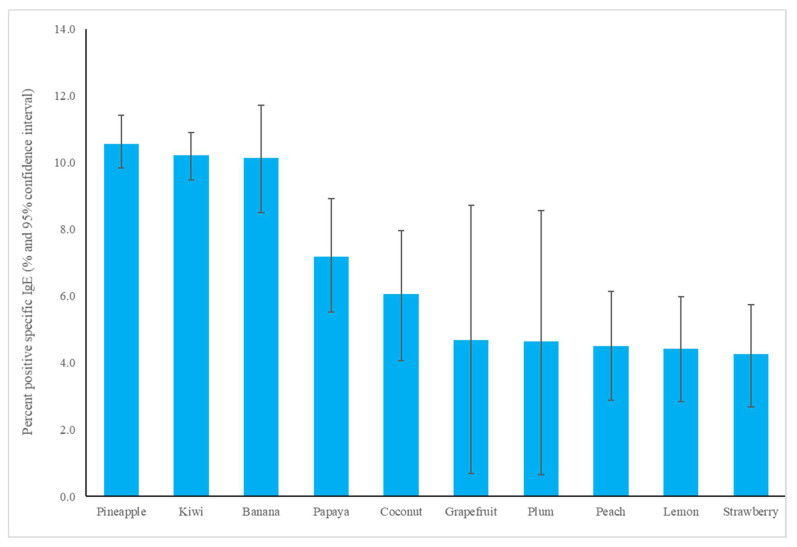
The specific immunoglobulin E positive rate of most common fruit allergens in children.

**Figure 2:**
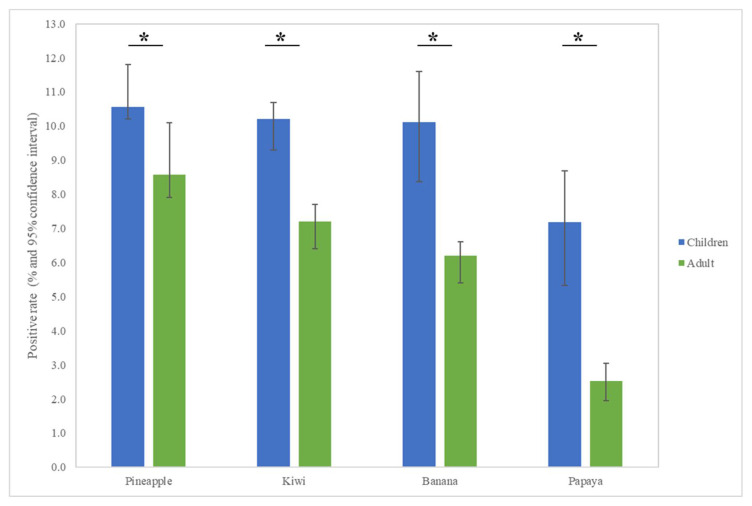
Allergen sensitization to fruits in adults and children displayed positive cases in each group. The results show statistically different sensitization rates between adults and children. * *p* < 0.05.

**Figure 3:**
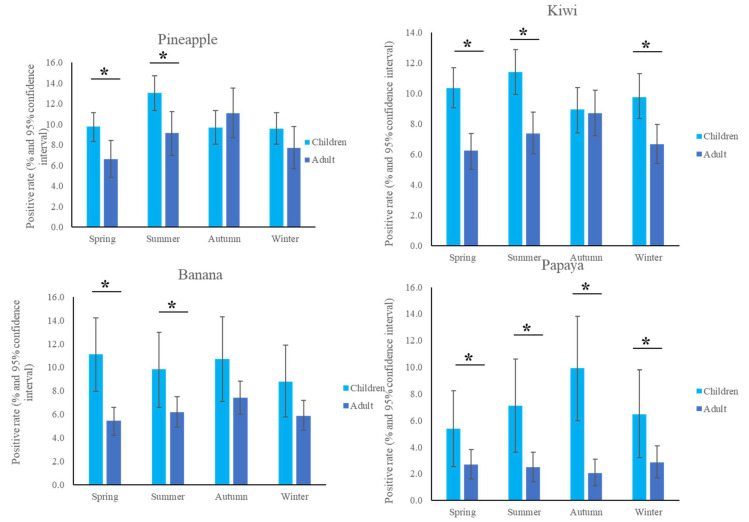
Positive sensitization rates to the top four fruits in the different seasons. * *p* < 0.05.

The most common fruit sensitizations in children were pineapple (specific antibodies detected in 10.6% of subjects), kiwi (10.2%), banana (10.1%), and papaya (7.2%) (Figure 1). For the purposes of this study, patients under the age of 18 years were defined as children, while those older than 18 years old were defined as adults. The fruit sensitization rate was higher in children than in adults (pineapple, *p* = 0.004; kiwi, *p* < 0.001; banana, *p* < 0.001; papaya, *p* < 0.001) (Figure 2). In the largest number of subjects, 628 positive tests for pineapple were obtained in children, accounting for a 10.6% (628/5951) positive rate, with the highest prevalence in summer (*p* = 0.003, Figure 3), although pineapples were produced in all seasons. The positive rates of sIgE for pineapple, kiwi, banana, and papaya were similar in both females and males (*p* > 0.05). Detailed data and the number of patients tested for each top allergen on sensitizing fruits are presented in Table 1. Younger age was significantly associated with sensitization to fruits in children (for kiwi 5.1 ± 3.8 and 5.8 ± 4.3 years old, *p* < 0.001, for banana 4.9 ± 4.5 and 6.9 ± 5.5 years old, *p* < 0.001, and for papaya 4.7 ± 4.3 and 7.1 ± 5.5 years old, *p* < 0.001) (Table 1). In contrast, no significant difference of age between negative and positive sIgE for pineapple was found. In other words, even in older children, the positive rate of pineapple allergens was high. The mean total IgE level and logarithmically transformed total IgE were significantly higher in pediatric patients with positive sIgE for pineapple, kiwi, banana, and papaya than in pediatric patients without (*p* < 0.001) (Table 1).

The analysis of the log IgE values of pineapple with significant differences for positive vs. negative sIgE allowed us to determine the optimal cutoff value of 2.2 (IgE 158.5 U/mL) with excellent sensitivity (0.9) and modest specificity (0.5) (Table 2). The analysis of the log IgE of kiwi with significant differences for positive vs. negative sIgE enabled us to determine the optimal cutoff value of 2.7 (IgE 501.2 U/mL) with modest sensitivity (0.7) and fine specificity (0.7). The analysis of log IgE for sIgE positive against banana vs. negative children obtained the optimal cutoff value of 2.5 (IgE 316.2 U/mL) with the high sensitivity (0.8) and modest specificity (0.6). With the sIgE for papaya, a log IgE of 2.4 (IgE 251.2 U/mL) appeared to be the optimal cutoff point for predicting the positive response, providing a sensitivity of 0.8 and a specificity of 0.6. The lowest cutoff value for the negative and positive sIgE for fruits was the log IgE of pineapple (IgE 158.5 U/mL) (Table 2).

Common risk factors of fruit sensitization in children include dermatitis, concurrent asthma/dermatitis, and dermatitis/rhinitis (Table 1). Clinical appearance among children with positive sIgE for pineapple showed higher rates of dermatitis (*n* = 302, 48.1%, *p* < 0.001), asthma combined with dermatitis (*n* = 124, 19.8%, *p* < 0.001), dermatitis combined with rhinitis (*n* = 270, 75.4%, *p* < 0.001), and asthma combined with dermatitis and rhinitis (*n* = 116, 18.5%, *p* < 0.001). Regarding the above clinical appearance and sIgE, children with positive sIgE for kiwi, banana, or pineapple have higher rates of dermatitis, asthma/dermatitis, dermatitis/rhinitis, and asthma/dermatitis/rhinitis than those with negative sIgE (*p* < 0.05). Children with positive sIgE for bananas have higher rates of asthma combined with rhinitis (*n* = 52, 38.8%, *p* = 0.010).

Among the abundant tropical fruits surveyed in Taiwan, some had lower sensitizations for children (2.3% positive specific antibodies of orange, *p* < 0.001 between pineapple and orange; 2.3% of mango and 2.1% of melon) (Supplementary Table S1). We analyzed allergens to pineapple, kiwi, banana, and papaya in children from the southern branch (Chiayi and Kaohsiung) and the northern branch (Keelung and Linkou) of CGMH and found no statistical difference between the north and south branches (*p* > 0.05 using χ^2^ tests, Supplementary Figure S1).

## 4. Discussion

To the best of our knowledge, this large-scale database study is the first to investigate fruit sensitization-based sIgE tests in Taiwanese children. As a result, we found that fruit sensitization was significantly associated with various factors including age, IgE, and clinical presentations. Applying judicious testing may be helpful in younger patients with symptoms indicating clinical allergy and higher total IgE who were likely to have fruit sensitization. The most important approach is to not indiscriminately remove tolerable fruit from one’s diet simply because they are related to a mild reaction [15]. A comprehensive examination of molecular allergies could assist physicians in deciding the most relevant allergen-specific immunotherapy [2,5,23].

The top four fruits with positive sIgE were all locally grown tropical fruits from Taiwan that were highly ingested by Taiwanese. Since the positive rates of challenge tests for pineapple and kiwi were the highest among fruits, the sensitization of these particular two needs special attention [16]. Of those, kiwi is imported, and the positive rate of sIgE in many countries around the world has been found to be relatively high [16]. Kiwi fruit is suspected of not only acting as a trigger for allergy symptoms but also increasing the risk of sensitization to other allergens, such as chickens [2,24]. Pineapple-induced oral allergy syndrome (OAS) is attributed to the presence of pan-allergen profilin, Ana c 1, whereas systemic IgE-mediated hypersensitivity is due to Ana c 2 (a major allergen, bromelain). Among 267 Mexican children aged 6–15, pineapple was identified as the most common food associated with OAS [25]. In another study, 1431 African school children aged 5–16, 2% were found to be sensitive to pineapple by the skin prick test [26].

Age and total IgE but not sex were significant risk factors for positive sIgE against fruits. The risk of sIgE was higher in allergic diseases among teenagers, and the sIgE of food was the second-highest among allergic diseases in Taiwanese children, second only to dust mites [27]. The reason for the higher positive rate of fruits in younger children may result from children’s immunologic and gastrointestinal immaturity, such as gut barrier and secretory immunoglobulin A [7]. Aging-related immune tolerance and modulation following long-term exposure to fruits lowered the positive rate of sIgE against fruits [3]. The high consumption of fresh fruits more than seven times a week was seen to be relevant to better cognitive functions in Taiwanese older adults [28]. There was not as much of a positive rate of fruits’ sIgE in adults as in children. These findings further stress the importance of a dietary pattern with fruits in adults [28]. However, in a study of more than 500 adults performed by dermatologists, it was found that the most patient-reported food allergy was induced by fruit and the most common diagnosis was urticaria [29]. We also checked that the positive rate of fruit sIgE was generally in line with the fruit sensitization rate among fruit-allergic individuals in the previous literature [2]. The number of clinical allergies with symptoms was much higher than those of people who tested positive [30]. Common sense suggests that since pineapple, banana, and papaya are tropical fruits which are produced in greater yield in summer, their intake in summer would also increase. Nonetheless, little evidence of a pattern of fruit consumption has been gathered over the seasons to support it in Taiwanese children [31]. Low sensitization rates of orange, mango and melon could be due to the low consumption of fruits, low allergenic potential or non-IgE-mediated mechanisms [8].

Pollen exposure plays a role in the prevalence of cross-reactive food sensitization mediated by IgE antibodies [32]. Both birch pollen and profilin-related food sensitization regularly occur in adults, and there is a gap between the percentages of children and adults demonstrating cross-reactivity with these allergens [33]. OAS with mild irritating local perioral itching symptoms is an allergic reaction that occurs after consuming fresh fruits and vegetables in patients allergic to pollen [15]. OAS can sometimes be combined with urticaria, angioedema, atopic dermatitis, respiratory presentations, anaphylaxis or gastrointestinal symptoms [33]. It is interesting that most adults (>90%) with positive specific IgE cross-reaction to lipid transfer proteins (LTP) developed clinical symptoms after the ingestion of sensitizing foods [33]. In areas with high exposure to mugwort or plane tree pollen, sensitization to pollen-derived LTP has been implicated as modifying the molecular-recognition spectrum by primary sensitization to pollen but has less been frequently seen in areas with low exposure and a positive rate to mugwort or plane tree pollen [34,35,36]. Peach allergy was found to be the leading cause of LTP-associated allergy and a precursor of allergy characterized by key components of cross-reactivity in adults, but was not the most common positive specific IgE in Taiwan [33]. Latex allergy has been related to allergies to specific foods including bananas, avocado and kiwi [15]. Unlike in Mediterranean countries or northern China, peach was not an important dietary ingredient in Taiwan, and the positive rate of pollen or latex in Taiwan was not high [27,34]. Therefore, the high positive rate of fruit in children was considered to be the primary plant source rather than the influence of cross-reactivity [30].

An important limitation of the present study is its selection bias in pediatric patients with higher IgE in the database. The reason may be that clinicians tended to check the specific IgE of the fruits for those patients with high IgE. Another limitation was the correctness of the diagnostic code by physicians in the database. Due to the lack of standard operating procedures and considering the low willingness to participate in challenge tests, no further skin prick or challenge tests could be performed [20,30]. In this report, we were unable to link specific IgE with contact history because some information was not provided in the unstructured database.

## 5. Conclusions

This study highlighted a higher sensitivity rate of fruit allergens to pineapple, kiwi, banana, and papaya in children inhabiting the Taiwanese region rich in tropical fruits. Leading complications of hypersensitivity in children were dermatitis and dermatitis combined with asthma or rhinitis. An analysis of sIgE tests revealed that the sensitivity rates for fruits were higher in children than in adults. Further prospective or national health and nutrition examination survey studies are warranted to confirm our findings and identify any clinical relationships.

## Figures and Tables

**Table 1:** Descriptive profiles and statistics of pediatric patients tested for fruit sensitization between positive and negative groups.

	**Negative Specific IgE to Pineapple**	**Positive Specific IgE to Pineapple**	***p***	**Negative Specific IgE to Kiwi**	**Positive Specific IgE to Kiwi**	***p***
Number	5323	628		6124	696	
Age (years)	5.4 ± 3.9	5.2 ± 3.4	0.126	5.8 ± 4.3	5.1 ± 3.8	<0.001 *
Sex			0.193			0.862
Female	2145	270		2478	284	
Male	3178	358		3646	412	
Total IgE (U/mL)	471.4 ± 673.7	1132.2 ± 1194.6	<0.001 *	442.5 ± 626	1305.8 ± 1359.5	<0.001 *
Logarithmic IgE	2.3 ± 0.6	2.8 ± 0.5	<0.001 *	2.3 ± 0.6	2.9 ± 0.5	<0.001 *
Asthma/rhinitis	53.1%	50.2%	0.159	48.5%	48.4%	0.956
Asthma/dermatitis	12.3%	19.8%	<0.001 *	11.2%	19.0%	<0.001 *
Dermatitis	29.9%	48.1%	<0.001 *	27.5%	48.1%	<0.001 *
Dermatitis/rhinitis	24.8%	43.0%	<0.001 *	22.1%	42.0%	<0.001 *
Asthma/dermatitis/rhinitis	10.9%	18.5%	<0.001 *	9.8%	17.7%	<0.001 *
Asthma	58.5%	54.9%	0.090	54.7%	53.7%	0.633
Rhinitis	86.7%	88.7%	0.150	84.4%	86.5%	0.148
	**Negative specific IgE to banana**	**Positive specific IgE to banana**	**p**	**Negative specific IgE to papaya**	**Positive specific IgE to papaya**	**p**
Number	1190	134		827	64	
Age (years)	6.9 ± 5.5	4.9 ± 4.5	<0.001 *	7.1 ± 5.5	4.7 ± 4.3	<0.001 *
Sex			0.472			0.392
Female	468	57		292	26	
Male	722	77		535	38	
Total IgE (U/mL)	470.5 ± 678.1	1287.3 ± 1927	<0.001 *	422.4 ± 601.4	1165.7 ± 1001.4	<0.001 *
Logarithmic IgE	2.3 ± 0.6	2.9 ± 0.5	<0.001 *	2.2 ± 0.7	2.9 ± 0.5	<0.001 *
Asthma/rhinitis	28.1%	38.8%	0.010 *	30.2%	32.8%	0.665
Asthma/dermatitis	6.1%	13.4%	0.002 *	5.2%	18.8%	<0.001 *
Dermatitis	21.5%	38.8%	<0.001 *	15.7%	51.6%	<0.001 *
Dermatitis/rhinitis	10.7%	23.1%	<0.001 *	10.6%	35.9%	<0.001 *
Asthma/dermatitis/rhinitis	4.3%	10.5%	0.002 *	4.2%	14.1%	0.003 *
Asthma	41.6%	50%	0.062	37.7%	53.1%	0.015
Rhinitis	64.3%	69.4%	0.240	73.4%	65.6%	0.178

* *p* < 0.05.

**Table 2:** Children with positive specific immunoglobulin E (sIgE) could be distinguished from those without by logarithmic IgE.

	log IgE			
	Cutoff	Sensitivity	Specificity	IgE U/mL
Pineapple	2.2	0.9	0.5	158.5
Kiwi	2.7	0.7	0.7	501.2
Banana	2.5	0.8	0.6	316.2
Papaya	2.4	0.8	0.6	251.2

## Data Availability

The data are available from the author upon request.

## Supplementary Materials

The following supporting information can be downloaded at: https://www.mdpi.com/article/10.3390/children9040470/s1, Figure S1: The positive sensitization rates of the four major fruits among children in the Chang Gung Memorial Hospital (South Branch, Chiayi and Kaohsiung; North Branch, Keelung and Linkou); Table S1: The numbers of patients investigated for fruit sensitization tests and the positive percentage.
