# Supplementary material for: Allergen Tests of Fruit Sensitization Involving Children with Allergic Diseases
Source: Children (Basel). 2022 Mar 28;9(4):470. doi: 10.3390/children9040470 (PMC9025653; doi:10.3390/children9040470)
Supplement: Supplementary file 1 [file children-09-00470-s001.zip › children-1622106-SI.pdf]

Supplementary Table S1: The numbers of patients investigated for fruit sensitization tests and the positive percentage.

	Children			Adults		
	Positive sIgE (Number)	Percent of Positive sIgE (%)	Total Tests	Positive sIgE (Number)	Percent of Positive sIgE (%)	Total Tests
Pineapple	628	10.6	5951	234	8.6	2731
Kiwi	696	10.2	6821	428	7.2	5939
Banana	134	10.1	1324	334	6.2	5385
Papaya	64	7.2	892	79	2.5	3128
Coconut	34	6.0	563	12	23.1	52
Grapefruit	5	4.7	107	0	0.0	5
Plum	5	4.6	108	0	0.0	5
Peach	28	4.5	624	135	5.1	2655
Lemon	29	4.4	658	2	8.3	24
Strawberry	28	4.2	659	17	3.1	549
Pear	28	4.2	660	16	2.9	558
Watermelon	26	3.9	666	26	3.3	783
Apple	38	3.7	1039	79	4.8	1644
Citrus	9	3.6	251	73	3.1	2389
Cherry	23	3.5	662	18	3.3	547
Orange	154	2.3	6688	145	2.5	5771
Mango	146	2.3	6385	119	1.9	6214
Melon	133	2.1	6309	143	6.1	2329
Grape	18	1.8	1017	24	17.1	140

sIgE, specific immunoglobulin E.

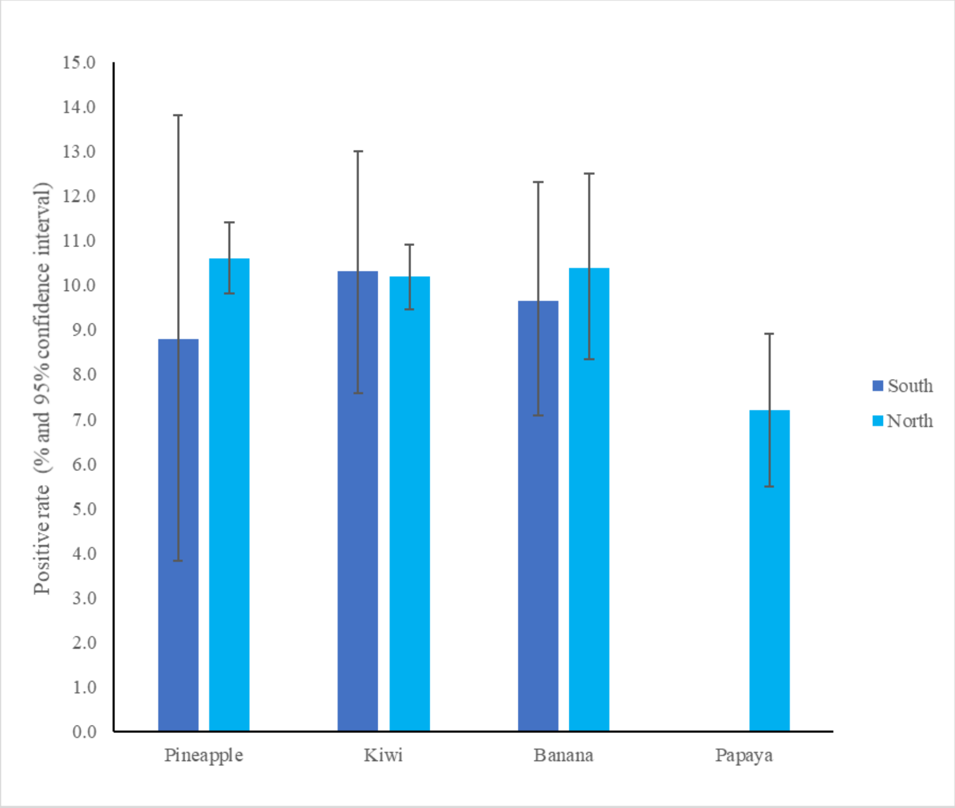

Figure S1. The positive sensitization rates of the four major fruits among children in the Chang Gung Memorial Hospital (South Branch, Chiayi and Kaohsiung; North Branch, Keelung and Linkou). A child in the South branch was tested for papaya allergen and the result was negative.
